# ERP Response Unveils Effect of Second Language Manipulation on First Language Processing

**DOI:** 10.1371/journal.pone.0167194

**Published:** 2016-11-28

**Authors:** Elvira Khachatryan, Flavio Camarrone, Wim Fias, Marc M. Van Hulle

**Affiliations:** 1 Laboratory for Neuro- & Psychophysiology, Department of Neurosciences, KU Leuven, Leuven, Belgium; 2 Neurology Department, Yerevan State Medical University, Yerevan, Armenia; 3 Department of Experimental Psychology, Ghent University, Ghent, Belgium; Leiden University, NETHERLANDS

## Abstract

Lexical access in bilinguals has been considered either selective or non-selective and evidence exists in favor of both hypotheses. We conducted a linguistic experiment to assess whether a bilingual’s language mode influences the processing of first language information. We recorded event related potentials during a semantic priming paradigm with a covert manipulation of the second language (L2) using two types of stimulus presentations (short and long). We observed a significant facilitation of word pairs related in L2 in the short version reflected by a decrease in N400 amplitude in response to target words related to the English meaning of an inter-lingual homograph (homograph-unrelated group). This was absent in the long version, as the N400 amplitude for this group was similar to the one for the control-unrelated group. We also interviewed the participants whether they were aware of the importance of L2 in the experiment. We conclude that subjects participating in the long and short versions were in different language modes: closer to monolingual mode for the long and closer to bilingual mode for the short version; and that awareness about covert manipulation of L2 can influence the language mode, which in its turn influences the processing of the first language.

## Introduction

One no longer needs to be perfect in two languages or to have acquired those languages in early childhood to be called bilingual. According to the currently most adopted definition, any person who uses more than one language on a daily basis can be considered bilingual [1]: neither proficiency level nor language domain (reading, listening, writing, speaking) is specified. For example, even if a person communicates mainly in one language (mother tongue), it is not uncommon that the main source of reading is in another language. That person still can be considered bilingual due to the daily use of two languages. When person prefers one of the languages over the other he/she is regarded as an unbalanced or dominant bilingual. In most cases, the preferable language is the mother tongue. As over 50% of the world’s population can be regarded bilingual, with the great majority being unbalanced, an increased interest is observed in investigating the unbalanced bilingual brain, the representation of languages in it and their interaction.

The interference of the mother tongue or dominant language (L1) while performing an overt task in the later acquired second (in most cases weaker) language (L2) was repeatedly shown in terms of the event-related potential (ERP–EEG response time locked to the target stimulus) [2–4] with the mechanisms of subconscious translation [4], competition [2], and so on. This technique is particularly appreciated for its excellent temporal resolution [5], in particular when investigating language processing, as the processing of language components (lexical access, phonology, semantics, syntax) develops over milliseconds [6]. The main ERP component attributed to processing of semantics in both L1 and L2 is the N400 [7,8]: a negative going EEG potential starting from around 300 ms after presenting the stimulus of interest and lasting till 500 ms post-onset. This potential is believed to reflect the processing of a potentially meaningful (linguistic in both L1 and L2 and non-linguistic) stimulus. The N400 is also believed to be an electrophysiological marker of the semantic priming effect, the facilitated processing of a word when preceded by a related word [8]. The priming effect is traditionally demonstrated by a faster response time (reaction time—RT) to the word, which was preceded by the related word [9]. The modulation (decrease) of the N400 potential in response to semantic priming, in one’s both first and second languages, and the interference of one’s first language while processing the second was repeatedly shown, especially for dominant bilinguals [2,10,11].

Unlike interference of L1 in L2 processing, the reports on the opposite effect (interference of L2 in L1 processing) are still ambiguous. A few behavioral [12–14] and electrophysiological/neuroimaging [15,16] studies claimed that, in the case of task performance in a purely L1 context, L2 does not influence L1 processing in either comprehension [12,13] or production [14]. On the other hand, there is also evidence in favor of L2 interference in L1 processing [17,18].

There are only few electrophysiological (ERP) studies that investigate this issue [15,16,18,19]. However, here also the reports were not unequivocal. Midgley et al., 2008 investigated the effect of cross language neighbors on the N400 potential in French English bilinguals in both French and English contexts. Though the effect of L1 on L2 processing was significant, the inverse effect (L2 on L1 processing) was much lower and only marginally significant. In another study [18], early balanced Welsh English bilinguals were performing a letter counting task in one of their languages in a semantic priming paradigm. Here, the authors observed the effect of semantic relatedness (less negative N400 potential) between word pairs for both languages irrespective of task language. These two electrophysiological studies advocate the interference of L2 while processing L1. However, note, that for Martin et al., 2009, L1 = L2, as their subjects were early balanced bilinguals.

Unlike Martin et al., 2009; and Midgley et al., 2008; Rodriguez-Fornells et al., 2002 did not observe any interference in terms of the word frequency effect [20] while their balanced, early Catalan Spanish bilinguals were performing a lexical decision task in the other language neither by electrophysiological nor by neuroimaging techniques. Therefore, they concluded that the competing items from the non-target language are inhibited before semantic information becomes available. The same conclusion was drawn in Hoversten et al., 2015. They assumed that in their Spanish English bilinguals the language membership information was available earlier than the information on conceptual representation of a word. This gave subjects opportunity to partially inhibit the access to semantic information on non-target language [16]. The difference between the outcomes of these studies does not account for the difference in complexity of the tasks, neither the presence of one or both languages in the experimental paradigm: in the case of Martin et al., 2009 a low level task such as letter counting was used and the L2 interference in L1 was observed, whereas in the case of Rodriguez-Formells et al., 2002, where words from both languages were presented, it was not observed. This difference could be explained by the concept of language mode. It is described as the state of activation of a bilingual’s languages and the language processing mechanisms at a given moment in time [21]. According to Grosjean, 1994, when a person is in monolingual mode, the ‘non-active’ language is deactivated (but not completely) and the other language is fully activated, which he called the ‘base language’. The concept of language mode was proven to be true for both production [22] and comprehension tasks [23]. For instance, Dunn & Fox Tree [23] showed that reaction time (RT) for rejecting non-words in lexical decision task was longer for English-Spanish bilinguals, when they were in bilingual rather than monolingual mode. Furthermore, they observed a positive correlation between RT and level of activation of the subject’s non-target language (the one that was not important for task performance, in this case, L1). Dewaele & Wei [22] studied a large number of multi-linguals (more than 2000) and showed the importance of a multilingual environment for the so-called ‘code switching’, which can be described as a language switch during the course of conversation. They concluded that in a bilingual environment, when both languages are active, individuals are more inclined to switch between languages. Therefore, one could say that the bilingual’s state can vary along the language mode continuum, where the monolingual and the bilingual modes are the extremes of a continuum. The position on this continuum depends on a number of factors, such as environment, task of experiment and proficiency level [21]. Soares & Grosjeans [24] suggested that bilingual individuals even in monolingual mode, cannot suppress their second language. In their study, they presented sentences from both languages in a blocked experimental design, therefore, they could not be sure whether their subjects were indeed in monolingual language mode. As mentioned before, a number of factors can influence a subject’s language mode, hence, we can assume when the subject is aware that the experiment is about bilingual abilities, he/she will switch to a more bilingual mode. We are going to address the question of language mode by considering an important, yet underestimated experimental issue: the subject’s awareness of a covert manipulation of L2 (such as in our case use of homographs in stimulus list) as a factor that can influence the subject’s language mode and the N400 response dependency on language mode. Surprisingly in only one behavioral study ([17], experiment 3) subjects were explicitly asked about their awareness of a covert manipulation or their general perception of the experiment. It could very well be that subjects noticed some L2 related pattern in an explicitly all in L1 experiment, which in turn could influence the subject’s language mode and, hence, the outcome of the experiment.

We hypothesize that among the factors that can affect language mode, and hence the level of L2 interference on L1 processing is the subject’s awareness of the importance of his/her L2 knowledge in the experiment or the awareness of a covert L2 related pattern/manipulation in the experimental stimulus list. We assume that the position on the language mode can shift even due to a hunch of an importance of L2 knowledge in the experiment. Furthermore, depending on language mode, the N400 pattern in response to the stimulus might be different.

In the current study, we test this question in highly proficient, though still unbalanced late bilinguals, not immersed in their second language environment. In order to address the issue of subject’s awareness, we conducted two ERP experiments: one with few and another with many distractor trials (called the short and long experimental versions, respectively), but using the same semantic priming task, target stimulus list and instructions. In the former, the subjects noticed the L2 manipulation of our experiment, hence paying more attention to their L2, therefore being in the intermediate language mode. In the long experimental version, many distractors were used to mask the purpose of the experiment (L2 manipulation), hence, making them believe that the experiment is designed to assess their L1 processing. This helped us to keep the subjects closer to the monolingual language mode. Additionally, in order to be sure about language mode subjects were in, we explicitly asked our subjects about their perception of the experiment and their awareness of any covert manipulation of L2. Finally, we provide a descriptive analysis of the recorded N400 potential to shed light on the mechanisms of L2 interference during L1 processing.

## Methods and Materials

### Participants

19 (11 female) healthy Dutch-English bilingual undergraduate and graduate students from KU Leuven participated in the long and 19 (12 female) in the short version of the experiment with an average age of 22.53 (ranging between 18 and 28) and 24.12 (ranging between 21 and 31) years old respectively.

Four out of 19 subjects for the long version and 6 out of 19 subjects for the short version were left handed. As the post-experimental questionnaire showed, none of the subjects was early bilingual: none of them acquired their L2 before age 6. They had no diagnosed neurological or psychiatric disability and were not on psychotropic medication. All participants had normal or corrected to normal vision. Participants were recruited via flyers and posters. Although their knowledge of English (L2) was not tested beforehand, in order to avoid providing them with a hint concerning the value of their knowledge of English for our experiment, we were aware that our subjects had a relatively high proficiency in English because they were graduate or undergraduate students that regularly or exclusively use course material in English. Furthermore, in the Flemish part of Belgium, where Dutch is the native language, media communication originally in English has Dutch subtitles instead of voice-overs. However, despite the prominent presence of English, our subjects were L1 dominant bilinguals as their main language of communication is Dutch. The study was conducted according to the ethical approval obtained from the ethical committee of our university hospitals, UZ Leuven (Commissie Medische Ethiek van de Universitaire Ziekenhuizen KU Leuven). Prior to the experiment, all subjects signed the informed consent form.

### Materials

For both experimental versions (long and short), the same four groups of experimental stimuli were used. The experiment was designed as a semantic priming task and presented as all in L1 (Dutch) experiment: both words of word pairs were presented in Dutch only, though a covert manipulation of L2 (English) was performed (in the processing of one of the stimulus groups L2 knowledge might play a significant role). In total, 60 semantically and/or associatively related and 60 unrelated Dutch word pairs were used. The same prime word (first word in the word pair) was paired once with a related target word and a second time with an unrelated one. In 30 word pairs, the prime word was an inter-lingual homograph: a word with the same lexical, but with different semantic representations in two languages (e.g. the word ‘angel’ means ‘sting’ in Dutch). We considered four stimulus groups (Table 1). The homograph unrelated group consisted of unrelated word pairs in Dutch with the inter-lingual homograph as prime word and the target word chosen in such a way that it was associated to the English meaning of the homograph (‘angel → vleugels’, meaning ‘wings’ in Dutch). The homograph related group consisted of related word pairs in Dutch with the homograph as a prime word, thus, with the target word associated to the Dutch meaning of the homograph, (e.g.–‘angel → bij’—meaning ‘honeybee’ in Dutch). The other two groups were control Dutch word pairs with and without associations between prime and target words (control related–‘hond → poes’: ‘dog → cat’ and control unrelated–‘hond → tafel’: ‘dog → table’, respectively). Moreover, in the long version of the experiment (for details see section Experimental procedure), we added 28 related and 28 unrelated control word pairs as fillers and 4 additional blocks of a semantic priming task to minimize the possibility that the subject will pay attention to the word pairs associated in English.

**Table 1:** Definition of stimulus groups. FAS refer to the portion of subjects that answered with that particular word in response to the presented prime word, + to presence and -- to absence of associations between prime and target words.

Stimulus groups	Homograph use as prime word	Semantic relatedness between prime and target words—average and standard error of forward association strength (FAS)	
		English	Dutch
Homograph unrelated	+	+ FAS = 0.1386 (0.03)	--
Homograph related	+	--	+ FAS = 0.1436 (0.01)
Control related	--	--	+ FAS = 0.126 (0.01)
Control unrelated	--	--	--

Lexical characteristics of target words are presented in Table 2. Word frequency (WF) of the target words was checked using the Dutch SUBTLEX word frequency database [25]**.** The orthographic neighborhood size (OTAN) and the length of the target words were checked using CLEARPOND non-commercial software [26]. As the filler word pairs used in the long experimental version were excluded from the analysis (both behavioral responses and ERPs), their characteristics do not play any significant role and hence, were not considered here. Repeated measure Analysis of Variance (ANOVA) showed no significant difference between the following stimulus characteristics between groups: WF (F (3, 119) = 0.45, p = 0.7144), length (F (3, 119) = 0.93, p = 0.4277), OTAN (F (3, 119) = 2.03, p = 0.114). For the full list of target stimulus word pairs see supplementary material (S1 Table).

**Table 2:** Lexical characteristics of target words for each stimulus group presented as means with standard deviation between brackets.

Stimulus groups	Word frequency	OTAN	Length
Homograph-unrelated	77.47 (122.22)	5.3 (5.96)	5.87 (1.98)
Homograph-related	173.46 (444.94)	4.23 (3.72)	5.6 (1.54)
Control-related	136.35 (239.43)	7.37 (5.29)	5.17 (1.7)
Control-unrelated	178.75 (551.88)	6.03 (5.01)	5.43 (1.38)

The values of forward association strength (FAS) for word pairs from control related (related group, Dutch control prime) and homograph related (related group, homograph prime) groups were taken from the word association database that is a part of the lexicon project ran by the Psychology Department of KU Leuven [27]. Repeated measure ANOVA showed that the defined groups did not differ in terms of FAS (F (1, 29) = 0.79, p = 0.3763). The FAS values for the word pairs from the homograph unrelated group were taken from the Edinburgh Associative Thesaurus (EAT) (association strength database for English words) [28].

### Single trial presentation

At the beginning of each trial, a cross appeared on the screen for a random duration between 500 and 700 ms, indicating that the subject should refrain from eye blinks or eye movements and fixate on the cross. Immediately after the cross disappeared, the prime word was shown for 300 ms. The target word was presented following the prime word for 300 ms with a jittered inter-stimulus interval of 300 ms on average (200 to 500 ms). Both words were presented in white Times New Roman font on a black background. The angular size of the word was around 4°. After the presentation of the target word, a blank screen appeared for 1000 ms, during which the EEG response to the target word was recorded. Then a screen with a question mark and two boxes, one labeled ‘Goed’ (‘good’) and another ‘Fout’ (‘wrong’) was displayed. The subject should indicate whether he/she thinks there is an association between the words of the word pair in his/her mother tongue (Dutch) by pressing one of two mouse buttons (left for the associated word pair and right for the unassociated one). This screen remained for 3 s or until the subject responded. Note, that the button-press response was delayed, beyond the time window the N400 ERP is expected, to avoid contamination with response-related ERPs [29]. After pressing the button, the subject received feedback on his/her response: ‘associeert‘ (related) for the left button press and ‘niet associeert‘ (unrelated) for right button press. The feedback did not reflect the correctness of subject’s response; rather it had a purpose to remind him/her about the role of each button. The response hand was counterbalanced across subjects: half of the subjects used the right hand and the other half the left hand. The explicit semantic association judgment task was chosen to increase the sensitivity of N400 potential in response to both explicit and implicit semantic relatedness between words, since it was shown [30,31] that although attention is not a necessary factor for elicitation of the N400, its presence increases the sensitivity of the latter. During this task, the subject should look for semantic (belonging to the same semantic category, e.g., ‘table—chair’) and/or associative (items from different semantic categories that are related to each other, e.g., cow—milk) relatedness between prime and target words. Though subjects were not explicitly instructed to ignore word pairs related in their L2, they were reminded on several occasions that the experiment and the task pertain to their mother tongue and that the goal of experiment was to study how the brain processes related items in their mother tongue. Hence, subjects were expected to judge word pairs accordingly and react based on word associations in their mother tongue.

### Experimental procedure

Each subject was tested in a sound attenuated, dimly lit room sitting on a chair in front of the LCD screen. The distance to the screen was around 70 cm. Prior to the experiment, eye movements and eye blinks were recorded using the aligned-artifact average (AAA) procedure described in [32] in order to remove in a later stage the artifacts caused by them. During the recording of eye movements, a white circle on a black background was moving vertically and horizontally starting from the center of the screen. Subjects were instructed to follow the circle with their eyes, without moving their head or blinking. During the recording of eye blinks, the same circle was coming down from the upper edge of the screen and was hitting the line in the middle of the screen. Subjects were instructed to focus on the center of the screen and blink every time the circle hit the line. They were specifically cautioned not to track the circle.

In both long and short versions, before starting the experiment, subjects were informed that the experiment is about how the brain perceives associations between words in their mother tongue (Dutch) and hence, during task performance, they should respond based on the associations believed to exist in their mother tongue. Nothing was mentioned about the words that can be related in their second language. Hence, normally the subjects should consider these word pairs as unrelated.

All word pairs were presented in a pseudo-random manner. As was mentioned earlier (section Materials), each prime word was presented twice during the whole experiment and its presentation counterbalanced so that half of the primes were first presented as a context in the unrelated word pair (stimuli from homograph unrelated and control unrelated groups), and the other half as a context in the related word pair (stimuli from homograph related and control related groups).

In the *short experimental version*, each prime word (both control word and inter-lingual homograph) was presented twice in the same block although maximally separated from each other to keep it more natural. This increased the probability of the subject to guess the covert manipulation of L2 or to pay attention to word pairs related in their L2. For the *long version*, the stimulus list was split into two blocks separated from each other by four distractor blocks on the same paradigm and task (semantic association judgment) with short breaks between blocks. In this case each prime word was presented only once within the same block so as to minimize, together with the filler control word pairs and blocks of distractors, the possibility of recalling those words or paying attention to the L2 manipulation.

Prior to the main experiment, each participant completed a short training session (six word pairs) on the same task, in order to familiarize the subject with the experimental paradigm and the task.

At the end of the main experiment, we asked each subject about their perception of the experiment: whether they noticed some specific language related pattern, which we did not mention in the instructions. After that, the participants performed another short block (English block) with the same semantic priming paradigm using stimuli from homograph unrelated group translated into English and another set of unrelated English word pairs in order to prove that homograph unrelated group can evoke a priming effect if presented in English. Therefore, if we do not observe any difference between control unrelated and homograph unrelated groups, we can assume that our results are due to the lack of the L2 interference in L1 processing and not because the associations (in English) for our homograph unrelated group were not strong enough to evoke semantic priming effect. After the EEG experiment, each subject received a list of homographs used in the experiment and was asked to indicate words for which the English meaning was unfamiliar with the aim of removing those words from further analysis.

The presentation of the experimental stimulus, as well as the session for eye calibration was performed using Matlab’s Psychophysics toolbox, non-commercial software [33]. The whole experiment with EEG recording, electrode cap mounting, explanation of the task, and short post-experimental survey lasted around two hours for the long and one hour for the short version. All instructions were presented on the screen in Dutch and the communication with the subjects before and during the experiment was also in Dutch.

### Electroencephalogram recording

The EEG recording was performed using 32 active Ag/AgCl electrodes (Bio-Semi ActiveTwo) placed according to the international extended 10–20 system. Additionally, six external electrodes were placed: one on the left and right mastoids, for further offline re-referencing. Other four were placed around the eyes, one on the upper and lower side of the left eye (vertical), and one near the external canthus of each eye (horizontal), for electrooculogram recording (EOG, bipolar recording). Except for these external ones, all electrodes were mounted in the electrode cap that was placed on the subject’s head. Conductive gel was applied in each of the electrode holes, as well as on the surface of the external electrodes to reduce electrode impedance. The signal quality was constantly inspected during recording. The signal was downsampled online from 2048Hz to 256 Hz.

### Data analysis

The EEG signal was re-referenced offline from the original common mode signal (CMS) reference to an average mastoids reference (AMR) and filtered using a 4^th^ order Butterworth filter in the range of 0.1 to 30 Hz. Choosing a reference method is very important, as, depending on the reference, the results might differ [34–36]. As the AMR is most commonly used in linguistic ERP studies [37,38] we adopted this referencing method so that we can compare our results with those of other N400 studies on language processing in the bilingual brain. Eye movement and blink artifact correction was performed with the method described in [32] using recorded EOG data. The EEG signal was segmented by defining windows starting from 100 ms prior to the onset of the stimulus of interest (target word) until 1000 ms post-onset. In order to remove trials with artifacts (residual eye movements, blinks and muscular artifacts), filtered epochs with an amplitude larger than +/-50 μV at channels of interest (C3, P3, Cz, Pz, C4 and P4) were discarded. These electrodes were chosen considering the centro-parietal distribution of the N400 potential.

In order to ensure that the obtained results are due to the interference of the L2 lexicon in processing of L1, we removed all trials containing homographs whose L2 meaning was unfamiliar to the subject. Though it is not usually done in ERP studies on bilingual language processing [39,40], we believe that by removing these trials we will obtain more objective results, as the interference would be much smaller if we include all the unfamiliar words. Furthermore, trials with wrong behavioral responses, no responses (in both versions) and filler trials in the long version were also excluded from the analysis (for the results with inclusion of abovementioned trials, see Appendix A). As we considered level of awareness of our L2 manipulation, hence, the subject’s language mode as the main condition for each experimental version, we also excluded subjects based on the outcome of our post-experimental questioning. For the remaining trials for each EEG channel, the baseline was removed using the average signal in the range starting from 100 ms prior to stimulus onset till target onset (0 ms). The N400 potential was measured as the mean amplitude from 350 to 500 ms after presentation of the target word. Additionally, we split the entire range of the N400 into three consecutive time windows with duration of 50 ms and further investigated their amplitude difference between stimulus groups.

To reduce the variance between 2 experimental versions, we converted, for each version separately, the N400 amplitude values into z-scores [41]. All processing was done in Python, using the NumPy and Pandas packages [42].

### Statistical analysis

The unstructured linear mixed effects model [43,44] was applied on ERP data to analyze the fixed effects of relatedness (Relatedness—related, unrelated), the use of homograph as a prime (Homograph—homograph, control), and their interactions (Relatedness × Homograph) for each experimental version. The mean amplitude from 350 to 500 ms post-onset (N400 potential), as well as the mean amplitudes in 50 ms intervals of the N400 were considered as dependent variables. We also included subject as random effect, in order to correct for the associations within each subject. Furthermore, the mentioned random effect neutralizes inter-subject variability; hence, the number of trials per subject does not play a significant role in the analysis. Tukey honest significant difference (Tukey HSD) test was used for post-hoc pairwise comparison. P values obtained from pairwise comparisons were corrected using Benjamini and Hochberg’s False Discovery Rate (FDR) method [45]. Satterthwaite approximation for degrees of freedom was used and the significance level of 5% was kept across the entire analysis. Statistical analysis was performed in R version 3.1.2 [46] using the Imer4 and ImerTest packages [47].

## Results

For both the long and short versions, after the main part of the experiment and before introducing the English block, we asked our subjects about their general perception of the experiment and if they noticed some linguistic manipulation that was not mentioned in the instructions. We considered the subject to be aware of the importance of his/her second language in the experiment when he/she mentioned the presence of associations in their second language (English). If they did not mention noticing word pairs related in their second language after the first question, we were explicitly asking them if they noticed that some words also could have a different meaning in English. We considered their response as a ‘guess’ if after our question they were recalling the presence of word pairs associated in English. Otherwise, if they were confirming the existence of these words, but mentioning them as unimportant for the experiment, we were considering their response as ‘no guess’.

For the long version, we excluded four subjects that indicated having noticed the L2 manipulation based on the previously mentioned criteria. For the short version, there were three subjects that did not notice the existence of word pairs associated in English; hence, we excluded them from the analysis.

After removing subjects that did not meet the requirements on guessing/not guessing, 15 subjects for the long and 16 subjects for the short version were included in the statistical analysis. Considering the results of our post-experimental questioning and our subject rejection criteria, we can claim that two experimental versions (short and long) that we used corresponded to the two different levels of subject’s awareness about L2 manipulation: high for short version and low for long version. In the short version of the experiment, subjects were aware about the presence of L2 manipulation (word pairs which meanings were related in English) in the experiment and some even guessed the purpose of the experiment (investigating how L2 influences L1 processing), hence we can consider them as closer to the bilingual end of the language mode continuum, while in the long version subjects did not pay attention to those word pairs, and were therefore unaware about their importance to the study, hence, we consider them as closer to the monolingual end of the continuum.

### Behavioral results

The average accuracy level of subjects’ performance on the semantic priming task was 0.9044 for the short and 0.926 for the long versions of experiment. It was calculated by taking the fraction of correctly responded trials with respect to the total number of trials. The average accuracy levels on individual stimulus group for each experimental version are presented in Table 3.

**Table 3:** Accuracy level of behavioral response for each stimulus group for short and long experimental versions listed as means and standard error (between brackets).

	Short version	Long version
**Homograph unrelated**	0.77 (0.02)	0.85 (0.02)
**Homograph related**	0.94 (0.01)	0.94 (0.01)
**Control related**	0.95 (0.01)	0.95 (0.01)
**Control unrelated**	0.94 (0.01)	0.95 (0.01)

An unstructured linear mixed effect model applied on the behavioral data obtained within each experimental version with the fixed effects of Relatedness, Homograph and their interaction (Relatedness × Homograph) and subject as random effect showed a significant effect of Relatedness and Homograph, as well as their interaction for both experimental versions (p < 0.001 in all cases).

When including the experimental version (EV—long, short) as a new fixed effect in the model for analyzing both versions jointly, it did not show a significance (F = 2.6, p = 0.12), though it’s interaction with Relatedness (EV × Relatedness) was significant (F = 7.15, p = 0.0076). This indicates that subjects from the short version were more often reacting incorrectly to stimuli from the homograph unrelated stimulus group (considering these trials as related) than subjects from the long version. The post-hoc comparison within each experimental version showed that in both cases, the performance on the homograph unrelated stimulus group was worse compared to the other stimulus groups (for all comparisons, p<0.01). The difference in performance for the homograph unrelated stimulus group might be explained by the so called “language borrowings” [48], which are words (or word-pairs/expressions), borrowed from the other language and regularly used also due to the absence of an equivalent translation (e.g., slot—machine). We believe that, as a result of language borrowing, our subjects (especially the ones of the long experimental version, which were closer to the monolingual language mode) were responding to word pairs from the homograph unrelated stimulus group as related ones.

When comparing behavioral responses between experimental versions per stimulus group using Tukey HSD test, only homograph unrelated groups were significantly different (F (1, 31) = 4.17, p < 0.05). For all other stimulus groups—p > 0.05. When calculating the effect sizes between them using the equation in Appendix B, we observed a larger effect size for the long experimental version for the homograph unrelated stimulus group (1.015) compared to the all other groups (0, 0 and 0.254 for the homograph related, control related and the control unrelated groups, respectively).

It is worth mentioning that when analyzing the behavioral data, we removed the responses to words that were unknown to the subjects, hence, we can assume that subjects in both experimental versions were performing the task by mainly following the instructions and responding to the word pairs of the homograph-unrelated group as unrelated (at least the ones from short version were trying to do so), even when the subjects from the short experimental version were aware about the associations present between the words in this group. Indeed, if this were not to be the case and subjects in the short experimental version would be reacting by relying on both English and Dutch associations, their performance accuracy for this stimulus group (homograph unrelated) after removing the unknown words, would have been much lower than it currently is.

Contrary to behavioral studies where subjects are requested to press a button immediately after seeing the target word (speeded response task), we implemented a delayed button-press, to avoid contamination of the N400 with response-related ERP components [29], but still to give the subject an explicit task to keep him/her alert. This implies that our reaction time data do not necessarily relate to the semantic priming effect, therefore, we chose not to include it into the analysis.

As a factor of subjects’ proficiency level, we calculated the average and standard error of number of unknown homographs for participants of each experimental version. It was 3.2 (0.49) and 4.1 (0.5) for short and long versions respectively. A Repeated Measure ANOVA showed no significant difference between number of unfamiliar homographs of experimental versions: F(1, 30) = 1.85, p = 0.18, hence, we can assume that the subjects from both our experimental versions had similar proficiency level in their L2.

### ERP results

Statistical analysis was performed on the three pairs of centrally (Cz and Pz) and laterally (C3, P3 –left and C4, P4—right) located electrodes. The data presented below comes from the central pair of electrodes. After presenting results on centrally located electrodes, we show the differences between the three mentioned pairs. The mean N400 amplitude was quantified for both L1 (4 groups presented in Dutch block) and L2 (2 groups in English block) stimulus groups. The choice of the electrodes comes from the known centro-parietal distribution of the N400 potential [31].

#### English (L2) block

In order to prove the validity of our stimulus list, we applied an unstructured mixed effects model on the means of N400 amplitude in the English block of both experimental versions taken together using subject as *random* effect and relatedness (related, unrelated) as fixed effect. For number of trials included in this analysis see Table 4.

**Table 4:** Final number of epochs used for analyzing English block results.

Stimulus Group	Number of epochs
**Related**	522
**Unrelated**	602

The outcome showed that the mean of the N400 amplitude in response to related English word pairs (translation of the homograph unrelated stimulus group) was significantly smaller than the one in response to unrelated word pairs (F = 19.27, p<0.0001). Thus, our English stimuli can elicit a semantic priming effect. Therefore, as it was predicted, if we do not observe any difference between homograph unrelated and control unrelated groups (section Dutch (L1) block: N400 potential (from 350 to 500 ms)—long version), we can conclude that it is due to the absence or lack of the L2 interference in L1 processing.

#### Dutch (L1) block: N400 potential (from 350 to 500 ms)

Here we present the statistical analysis of each experimental version for the centrally located electrode pair (Cz, Pz). The numbers of trials included for this analysis and the mean amplitudes of N400 potential for each stimulus group for the mentioned electrode pair are presented in Tables 5 and 6 respectively.

**Table 5:** Final numbers of trials per stimulus group for each experimental version in Dutch block.

	Experimental version	
Stimulus Group	Long version	Short version
	(15 subjects)	(16 subjects)
**Homograph unrelated**	249	267
**Homograph related**	291	317
**Control related**	305	357
**Control unrelated**	333	363

**Table 6:** means and standard errors (SER) of amplitudes of N400 potential in centrally (Cz, Pz) located electrode pairs.

Stimulus groups	Experimental version	Mean (SER)
Homograph unrelated	Short	-0.02 (0.061)
	Long	-0.086 (0.066)
Homograph related	Short	0.159 (0.057)
	Long	0.269 (0.055)
Control related	Short	0.174 (0.052)
	Long	0.163 (0.054)
Control unrelated	Short	-0.229 (0.049)
	Long	-0.186 (0.056)

**Fig 1:**
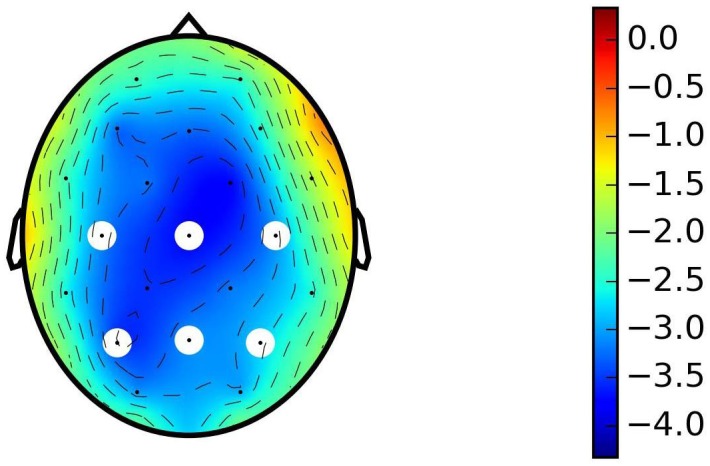
Scalp plot of N400 effect for control groups of both experimental versions taken together. The N400 effect is presented as a difference between unrelated and related target words. The mean of entire time window of N400 potential (350 to 500ms) is represented. The N400 in response to the control unrelated word pairs shows greater negativity compared to the control related word pairs with the centro-parietal spatial distribution. White dots represent the locations of electrodes of interest: C3, P3 —left, Cz, Pz—central, C4, P4 —right.

In both experimental versions the difference between amplitudes of N400 potentials for control groups (unrelated—related) showed similar negativity between 350 and 500 ms post-onset with centro-parietal spatial distribution (semantic priming effect) (Fig 1).

**Fig 2:**
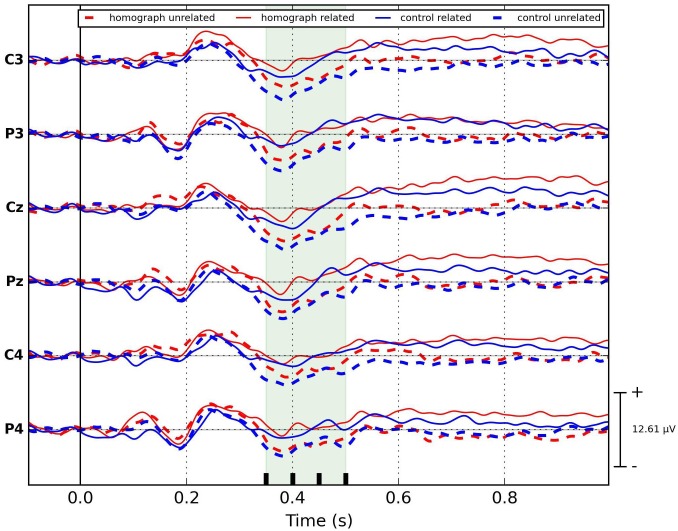
ERP plots for all stimulus groups for long experimental version. N400 time range is shaded. No priming effect is present for the homograph unrelated stimulus group.

*Long version* (Fig 2): the unstructured linear mixed model with fixed effects of Relatedness, Homograph and their interaction (2×2 design) applied on the means of N400 amplitude, showed a significant effect of Relatedness (F = 38.19, p<0.0001) but no significant effects of Homograph (F = 3.68, p = 0.055) or Relatedness × Homograph interaction (F = 0.008, p = 0.9). A post-hoc Tukey HSD pairwise comparison showed that the N400 potential in response to control unrelated group was significantly larger compared to the homograph related (F = 35.048, p<0.0001) and control related (F = 19.92, p = <0.0001) groups. Further, N400 potential in response to homograph unrelated group was significantly larger than the ones in response to homograph related (F = 17.53, p<0.0001) and control related (F = 8.67, p = 0.005) groups. No significant difference between homograph unrelated and control unrelated (F = 1.42, p = 0.234) or homograph related and control related groups (F = 2.27, p = 0.163) was detected. *In summary*, *we can say that in the long version of experiment*, *where the awareness about the L2 manipulation was minimal and the subjects were closer to the monolingual end of the language mode continuum*, *the amplitude of N400 potential was significantly larger (more negative) for unrelated (both homograph and control) groups compared to the related ones in monolingual context*.

**Fig 3:**
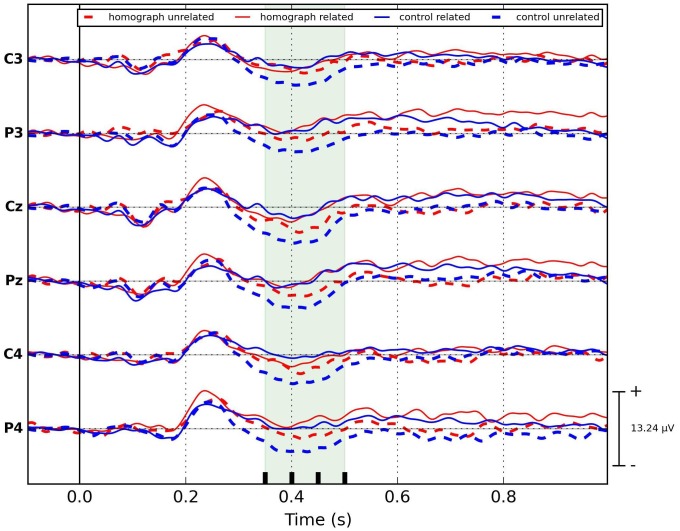
ERP plots for all stimulus groups for short experimental version. N400 time range is shaded. Significant priming effect is presented for the homograph unrelated stimulus group.

*Short version* (Fig 3): the unstructured mixed model (2×2 design), applied on the mean amplitudes of N400 potential with fixed effects of Relatedness, Homograph and their interaction, showed a significant effect of Relatedness (F = 29.43, p<0.0001) and Relatedness × Homograph interaction (F = 4.077, p = 0.044). The effect of Homograph was not significant (F = 3.31, p = 0.069). The post-hoc Tukey HSD test revealed that the mean amplitude of N400 for control unrelated group was significantly larger than for all other groups (homograph unrelated—F = 7.69, p = 0.012; homograph related—F = 27.55, p<0.0001 and control related—F = 32.4, p <0.0001). For the other groups, the mean N400 amplitude was significantly larger for homograph unrelated group compared to homograph related (F = 4.75, p = 0.036) and control related (F = 5.9, p = 0.022) groups. No significant difference between the N400 amplitudes of homograph related and control related groups was observed (F = 0.029, p = 0.866). *To summarize*, *for short experimental version*, *where subjects’ awareness about L2 manipulation was high and he/she was in the intermediate language mode (closer to the bilingual end of the continuum)*, *the largest N400 amplitude was for control unrelated group*, *followed by homograph unrelated group*, *which was larger than the ones for control and homograph related groups*. *The N400 amplitudes for the last two were equally small*.

When jointly analyzing the N400 amplitude data from both experimental versions, the inclusion of experimental version (EV—short, long) as fixed effect into the model did not show significance (F<1.0), neither did its interaction with any other previously mentioned factors (Relatedness, Homograph). It can be explained by the fact that in order to put the subjects from two experimental versions into the same conditions, we excluded the trials with incorrect behavioral responses from all statistical analyses (both intra and intergroup). Doing so, we factually decrease the existing difference between experimental versions. Hence, as the subject groups were different, the said precaution seems to be responsible for the absence of an effect of experimental version in the ERP data.

#### Dutch (L1) block: amplitudes of 50ms individual time windows (Figs 4 & 5)

**Fig 4:**
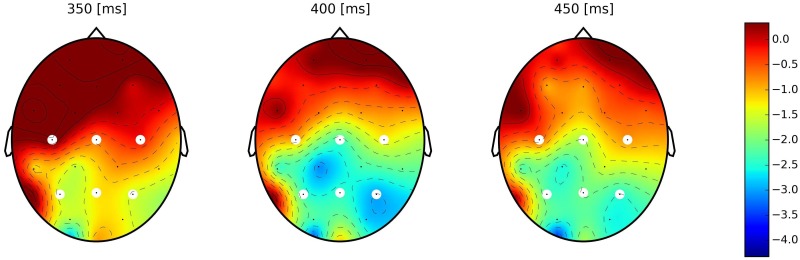
Scalp plots of difference between homograph unrelated and homograph related groups for short experimental version. Each scalp plot represents the average of 50 ms time window (from left to right: 350 to 400 ms, 400 to 450 ms and 450 to 500 ms accordingly). An increase in negativity (N400_hu_-N400_hr_) over time is observed.

**Fig 5:**
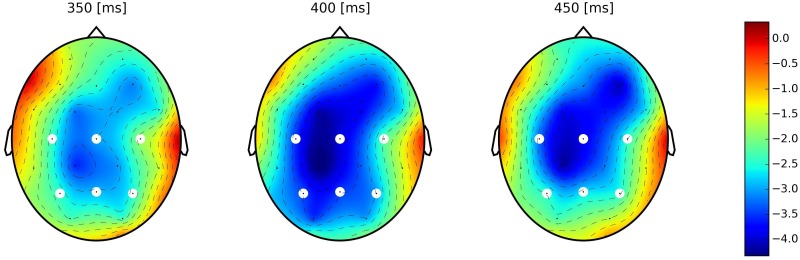
Scalp plots of difference between homograph unrelated and homograph related groups for long experimental version. Each scalp plot represents the mean of 50 ms time window (from left to right: 350 to 400 ms, 400 to 450 ms and 450 to 500 ms accordingly). A persistent negativity (N400 effect—N400_hu_−N400_hr_) is observed in all time windows.

In order to perform a detailed investigation of the N400 potential and to observe it’s development over time we uniformly split it into three consecutive intervals of 50 ms duration (indicated by vertical lines in the shading in Figs 2 and 3). We applied an unstructured linear mixed model on the mean amplitudes of these intervals in each experimental version with the inclusion of time window (TW– 3 level) as fixed effect in addition to the previously introduced fixed effects (Relatedness (2), Homograph (2)), hence, with the 2×2×3 design for each experimental version. Results showed significant effect of Relatedness: short version—F = 74.31, p<0.0001, long version—F = 92.28, p<0.0001; Homograph: short version—F = 8.15, p = 0.004, long version—F = 8.78, p = 0.003, and TW: short version—F = 14.68, p<0.0001, long version—F = 26.11, p<0.0001. From all interactions, the only significant one was the Relatedness × Homograph interaction for the short version only (F = 10.02, p = 0.0016).

Next, we investigated the effect of Relatedness, Homograph and their Relatedness × Homograph interaction on N400 amplitude in each individual 50 ms time window in each experimental version (2×2 design). Results are presented in Table 7.

**Table 7:** Effects of factors (R, H) and their interaction on the amplitude in individual 50ms interval of N400 potential for the centrally located electrode pair (Cz, Pz).

*Fixed effects*	*Experimental version*	350 – 400ms	400 – 450ms	450 – 500ms
		*F (p)*	*F (p)*	*F (p)*
**Relatedness**	*Short*	15.03***	32.37***	28.78***
	*Long*	28***	28.2***	36.7***
**Homograph**	*Short*	3.9*	3.24 (0.072)	1.41 (0.235)
	*Long*	4.48*	5.04*	0.47 (0.493)
**Interaction**	*Short*	5.74*	2.74 (0.098)	2.17 (0.141)
	*Long*	0.007 (0.93)	0.58 (0.446)	0.437 (0.509)

*—p<0.05

**—p<0.01

***—p<0.001

A post-hoc Tukey HSD test on the mean amplitudes within individual time window showed that, for the ***short version*** (Fig 4), the priming effect (smaller N400 amplitude compared to control unrelated group) for the homograph unrelated group was significantly stronger with effect size [49] of *0*.*24 μV* (for the formula of effect size calculation, see Appendix B) in the early N400 time window (350 to 400 ms) compared to later ones (400 to 450 ms and 450 to 500 ms) where effect sizes were *0*.*19* and *0*.*16* respectively. For this early window, there was no significant difference between both related groups and the homograph unrelated group (F = 1.58, p = 0.31 for control related group and F = 0.87, p = 0.42 for homograph related group). This changed at the end of the N400 range, as the potential in response to homograph unrelated group gradually became more negative and in the 450 to 500 ms time window, the N400 amplitudes of the control unrelated and homograph unrelated groups were not statistically different (F = 4.07, p = 0.053). In this experimental version, the control and homograph related groups were not different across the entire N400 interval (p>0.05 for all time windows).

In contrast, in the ***long version*** (Fig 5), N400 amplitudes in response to homograph unrelated and control unrelated groups did not differ statistically across the entire range of the N400 (p>0.05 for all windows). Furthermore, only in the 400 to 450 ms window, the N400 amplitude of homograph related group was significantly smaller (with amplitude difference of 0.164) than that of control related group (F = 4.6, p = 0.038). This can be explained by the fact that word pairs from homograph related stimulus group had a marginally higher forward association strength compared to control related group (namely FAS = 0.0176) albeit was not significant. Hence, this might lead to a small but significant strengthening of the priming effect for homograph related group in this short time window. As the stimulus list was completely the same for both experimental versions, the absence of this effect in the short version, in particular, the more negative N400 amplitude in response to the homograph related group might be explained by the inhibition present in this experimental version that probably affected stimulus groups that have a homograph as a prime.

When jointly analyzing both experimental versions, the unstructured linear mixed model applied to the mean amplitudes of 50 ms time windows of N400 potential with the inclusion of experimental version (EV—2 level) as fixed effect in addition to previously introduced effects of Relatedness, Homograph and time window (TW) in a 2×2×3×2 design, showed significant effects of Relatedness (F = 166.51, p<0.0001), Homograph (F = 16.95, p<0.0001) and TW (F = 38.13, p<0.0001). From interactions, the significant ones were Relatedness × Homograph (F = 4.375, p = 0.036) and EV×TW (F = 3.28, p = 0.037). This time, the Relatedness × Homograph × EV interaction was significant (F = 5.14, p = 0.023), unlike in previous analysis where time window was not included as fixed effect.

#### Dutch (L1) block: spatial distribution of N400 effect (channels C3, P3, C4, P4)

**Fig 6:**
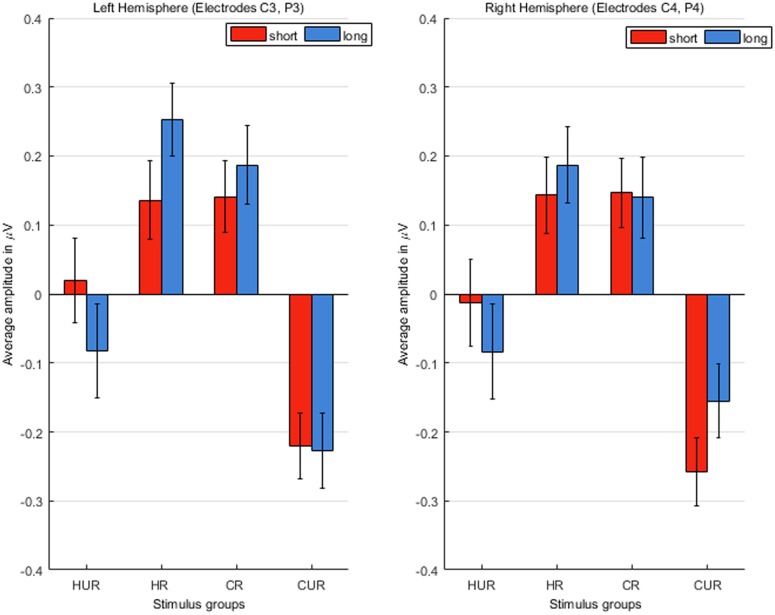
Means and standard errors of N400 amplitudes in laterally (C3, P3, C4, P4) located electrode pairs.

As differences in spatial distribution for L1 and L2 have been reported [50], we also checked (Figs 2 & 3) whether our ERP changes are lateralized toward one of the hemispheres or are more centrally located in the case of the covert manipulation of L2. To this end, we repeated statistical analysis (section Statistical analysis) on the mean amplitudes of N400 obtained from the laterally located electrode pairs (C3, P3 –left, C4, P4 –right). For means and standard errors of N400 amplitudes for these electrode pairs see Fig 6.

We observed that in laterally located electrodes (C3, P3, C4, P4) the priming effect for homograph unrelated group was stronger compared to centrally located electrodes (Cz, Pz) albeit for the short version only. In this case, the N400 amplitude was significantly larger only for control unrelated group compared to all other groups (for all comparisons p<0.005). No significant difference was observed between both related (control and homograph) and homograph unrelated groups.

In long experimental version, the difference between homograph unrelated and control unrelated groups was not significant for either of the electrode pairs—left, right or central (p>0.05 for all cases).

## Discussion

### L2 interference during L1 processing and non-selective lexical access

The current ERP study was designed to shed light on the divergence in previous reports on L2 interference while subjects perform a monolingual (L1) task. We conducted two studies with the same target stimulus list and experimental paradigm but using different presentation types (termed short and long experimental versions). Unlike the long version, in the short version the subjects became aware of the covert L2 manipulation. Here we observed a significant L2 (English) interference and some remaining priming of word pairs related in English even when subjects were informed that the associations were going to be in their mother tongue (Dutch) and behaviorally responded to word pairs associated in English as unrelated (we included only those trials into the analysis). Hence, there was no need for our subjects to activate both lexicons.

Our results from the short experimental version add credit to the hypothesis of non-selective lexical access [51]: a word presented in one of the subject’s languages activates its translation and related words in both languages independent of the experimental context (monolingual or bilingual). There are several models that are in line with this hypothesis in written word recognition, among them, the revised hierarchical model (RHM) [52], bilingual interactive activation (BIA) [53] and bilingual interactive activation plus (BIA+) [54] models. All these models claim the existence of a competition and active inhibition of competing words within and across languages. None of them mentions anything about a possible pre-activation or deactivation of the supposedly ‘non-active’ language. In our study, we showed that the interference mechanism of L2 while processing L1 operates by inhibition together with remaining priming effect from L2 in the short experimental version where the task requires the inhibition of the non-target language (L2 in this case) and a de-activation of the non-target language in the long experimental version. We assume that inhibition observed in the short experimental version is most probably a task-driven top-down process, and is a part of lexical selection. For the short experimental version, as our subjects were aware that items could be semantically related in their L2, the remaining priming effect from L2 was evidenced by the smaller N400 amplitude in response to the homograph-unrelated group compared to the control-unrelated group. At the same time, as our task required to consider word-pairs related in L2 (homograph-unrelated) as unrelated, the developed inhibition impedes the N400 in response to this group to reach the level of the two related groups. There is at least another sign of inhibition in the short experimental version: the absence of a difference between N400 amplitudes for the two (homograph and control) related groups, unlike in the long experimental version where the N400 amplitude for the homograph related group was smaller compared to the control related group, albeit for a short period of time only (400–450 ms). When comparing these N400 responses between experimental versions, as the same stimulus list was used (with marginally higher FAS for the homograph-related group compared to the control related group), we can attribute this difference to the effect of inhibition in the short experimental version. The said inhibition was also suggested in a behavioral study by De Groot et al. [55]. On the other hand, Dijkstra et al. [56] claimed that the absence of a RT difference between control items and homographs in lexical decision task was due to a similar processing of those two types of items with no inhibition present. The fact that those studies could not discern between inactive state and inhibition is probably due to the method used (only RT). When using the ERP technique and analyzing ERP amplitudes in short time-windows, we can suggest a possible mechanism: a priming and facilitation of related items in only the target language (L1 in our case) in our long experimental version and a remaining priming of items related in the non-target language (L2 in our case) and a task—related inhibition in our short experimental version. However, we hasten to add that, as we reached this hypothesis based on relative changes in N400 amplitude only, we acknowledge that more in-depth studies are needed in order to unveil the exact timing between the said processes.

### Awareness of covert manipulation and language mode continuum

As we probed the subject’s awareness about the covert manipulation of the presumably ‘non-active’ language, we can discuss our behavioral and electrophysiological results in the context of the putative influence of language mode [1,24]. Unlike the short experimental version, where a significant priming effect was observed for stimuli from the homograph unrelated group, no significant priming effect for this stimulus group was observed in the long experimental version. When questioning our subjects after the experiment, the ones that participated in the short version reported that they were aware of word pairs related in their second language, and even some of them guessed the purpose of the study (how L2 influences the processing of L1) and the role of their second language knowledge in the experiment. This also was reflected in the behavioral results, as here the subjects were more frequently responding incorrectly to trials from the homograph unrelated group than in the long version. In contrast, subjects from the long experimental version did not have a clue about the value of their second language knowledge in the experiment. The results from these two experiments by consequence show that even a hunch (intuitive guess) about second language manipulation during an explicitly monolingual task (in addition to other factors hypothesized by Grosjean [21]) can influence the outcome of the study.

**Fig 7:**
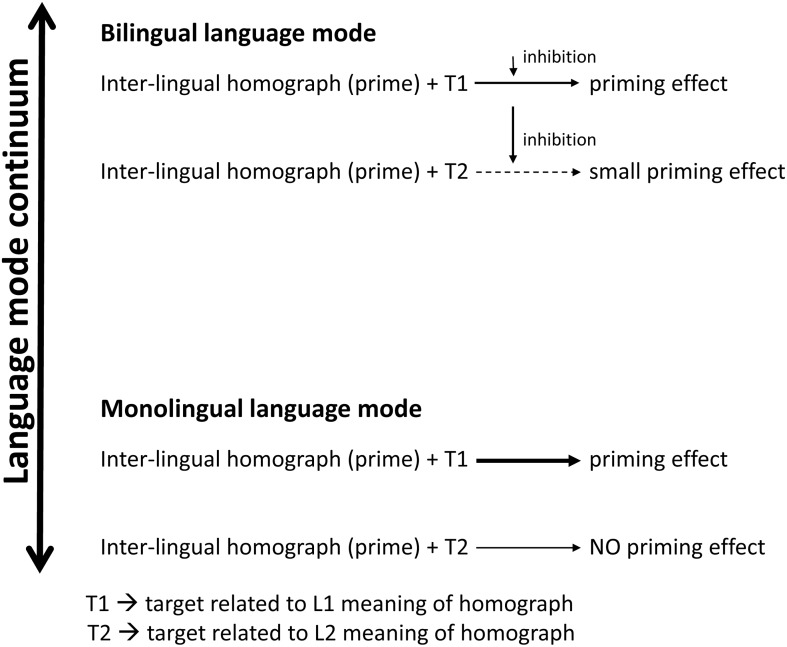
Schematic representation of semantic priming in bilinguals in different language modes.

We showed that, when the subject is unaware of the covert manipulation, which was the case in the long experimental version, the interference of L2 is not strong enough to be significant. These results can be explained when referring to the concept of language mode. Based on our post-experimental questioning, we can say that our subjects in the short version of the experiment, even when presented with the same instructions and experiment setting as for the long version, were closer to the bilingual mode (intermediate mode). So, instead of claiming that it is not possible to “shut down” the non-target language [55,56], we can assume that once this language is activated during the experiment, even due to the subject’s hunch of its importance (as it was the case in the short version of our experiment), it is no longer possible to “switch it off” even if the subject is instructed to perform the task in one language only (thus, there is no need to activate the other language). In contrast to this, in the long version, because of the multiple distractor trials, subjects did not pay attention to the word pairs related in English, hence they were much closer to the monolingual mode of the language mode continuum. Therefore, in this case we can assume that subjects’ L2 (English) was minimally active; therefore, it did not lead to significant difference between N400 amplitudes for homograph unrelated (related in English) and control unrelated groups. Note, that we do not claim our subjects to be in mono- or bilingual mode, but rather to be closer to one or the other mode. This assumption still does not go against the non-selective lexical access hypothesis the mentioned models are based on. Although lexical access indeed can be non-selective, the search within the lexicon could mostly occur among the words from the ‘active’ language. If lexical access would be selective, as claimed elsewhere [13], it would not be that easy to access both languages at the same time, based only on a hunch of the subject. The notion that words from different languages could be activated more or less depending on the subject’s position on the language mode continuum (even for a single integrated lexicon as described in BIA model for example [57]), would render the models of lexical access more complete and could explain the divergence in the outcome of several studies [13,15,18]. In Fig 7, we show a possible scheme of semantic priming in different language modes.

There is a pending question on the control of language mode in our experimental versions, as we assumed that the individual’s language mode can be discerned from their conscious self-report. Currently, there is no accepted method to evaluate an individual’s language mode during comprehension tasks. As we mentioned in the introduction, one behavioral study [17] investigated the individual’s language mode based on self-reports. In a group of studies [58,59], evaluating the phonological distinctions between a bilingual’s two languages they were setting the language mode by changing the language of instruction (but note, that in this case, they were studying two monolingual modes, L1 or L2). We are not aware of any event-related potential study that probed the subjects’ language mode or even considered it as a confounding factor. We were inspired by the hypothesis that subject’s knowledge about the importance of his/her bilingual abilities in the experiment can influence their position on the language mode continuum [21]. Whence, in our ERP study, we used the conscious self-report to evaluate subject’s language mode during comprehension task. Furthermore, the ERP pattern in our long experimental version was similar to the one of monolinguals, separation of stimulus groups into related versus unrelated word-pairs and the ones from the short experimental version showed the interference of second language in the first language processing in terms of a change in N400 amplitude in response to the homograph-unrelated group. Considering the ERP response is reflexive and not under the individual’s control, it gives us a strong argument in favor of covering non-declarative aspects of language mode testing. Therefore, we can assume that the conclusion on whether the subject is closer to the monolingual or bilingual end of the language mode continuum was justified and successful. Supportive evidence also comes from behavioral studies, as it was shown on several occasions (for review, see [60]) that keeping a highly proficient bilingual subject in monolingual mode is more difficult than in bilingual/intermediate mode. Whence, if our subjects from the short experimental version consistently reported on spotting word-pairs associated in their second language, we can safely assume, that they were closer to the bilingual end of the language mode continuum. In summary, even though we do not claim that awareness about a covert L2 manipulation (based on their self-report) is the only, or even the main factor that influences the subject’s language mode, we do advocate that this important, yet underestimated factor can influence the outcome of the experiment.

Most models of lexical access in the bilingual brain (BIA, RHM, model of selective lexical access, and so on) consider lexical access to be either “selective” or “non-selective”, hence, treating the question of selectivity in a yes/no manner. The Bilingual Model of Lexical Access (BIMOLA) is the only bilingual lexical access model that takes into account the individual’s language mode. It considers the existence of two independent, but at the same time interconnected networks (lexicons) and therefore introduces the concept of partial selectivity in lexical access, suggesting that subjects can activate their lexicons to some degree depending on the number of bottom-up (e.g., stimulus list) and top-down (e.g., linguistic environment and instructions) information. Our results are in accordance with this model. Spivey & Marian, 1999 [61] when conducting an eye-tracking study on Russian-English bilinguals found that the interference of English in Russian was larger than Russian into English when the subjects were immersed into an English speaking environment, even when it was their L2. They assumed that, when presented with a word in one language (it was an auditory presentation in their case), the subject automatically activates both mental lexicons in parallel, albeit one only partially, as the interference was less in one language compared to the other. Hence, in our short experimental version, we can also talk about partially selective lexical access: although the N400 amplitude in response to the homograph unrelated group was smaller compared to the control unrelated group, in the centrally located electrodes it was still larger compared to the amplitudes in response to the homograph related and control related groups. This, we assume, can be due to the presented instructions.

Unlike centrally located electrodes, for the lateral (both left and right) electrodes, no difference was observed between homograph-unrelated and the two related groups, albeit for the short experimental version only. This indicates a more profound priming effect for the lateral electrodes. The absence of such a divergence across electrodes for the long experimental version, stresses once again the notion that in language mode, closer to monolingual end of language mode continuum, indeed mainly one language is active, hence, no difference between the processing of homograph- and control- groups was observed across all pairs of electrodes. A less pronounced priming effect for homograph-unrelated group in the central electrodes can be due to an increased inhibition for those electrodes. Indeed, as the fronto-parietal network (for review see [62]) is considered responsible for cognitive control and also involved in second language processing [62], we assert that the observed inhibition in these electrodes is due to the top-down cognitive control.

The difference between inhibition and inactivation of a lexicon plays a key role in the current study, as we showed that depending on the language mode one or the other can occur. The inactive state (long experimental version, closer to monolingual mode) is a passive condition, whereas inhibition is an active process, guided by top-down processes, in our case, most probably the task requirements to pay attention to the mother tongue. Hence, we can assume that both theories about N400 formation (facilitation of the related word [63] and inhibition of the unrelated word [64]) are valid, albeit for different experimental versions, when subjects are using different strategies. Our current assumption can be linked to our data by observing the difference between homograph-related and control-related stimulus groups in the long experimental version, which is absent in the short version. This difference (albeit in the short time-range only) could be attributed to the slight (statistically not significant) difference in forward association strength (FAS) between the two stimulus groups (note, that this difference was common to both experimental versions). The absence of inhibition in the long experimental version, where subjects were not aware of their L2 manipulation that could have demoted the processing of the homograph-related group, resulted in a difference between the homograph-related and control-related groups with a smaller N400 amplitude in response to homograph-related group (though for a short time only), which is also observed in other monolingual studies with semantic priming [65]. On the other hand, in the short experimental version, where subjects were aware about their L2 manipulation this difference was absent, probably due to an active inhibition of L2, which was also observed to influence the homograph-related group rendering its processing more difficult.

Although few electrophysiological studies report on the interference of L2 while processing L1, to the best of our knowledge, we are the first to consider a semantic association judgment task for late dominant bilinguals not immersed in their second language. The use of semantic association judgment task allows us to fully access the conceptual representation of the lexical item, at the same time showing that even in that case it is possible to keep the subject close to the monolingual mode (as in the long experimental version). Furthermore, the current study is the first one to explicitly show that subjects can find out by themselves the relevance of their second language knowledge for experiment or notice the pattern in the experimental paradigm and as a result move along the language mode continuum where they will remain during the course of the experiment.

In sum, our results show the importance of the subject’s language mode during the experiment, as it might change when they become aware of a covert manipulation or change their general perception of the experiment, as there is a possibility that the subject might figure out the covert goal of the study. We showed that this can affect the experimental results and obtained effects (Fig 7). Furthermore, we showed that depending on the subject’s language mode, the mechanism of processing incoming lexical information might change, either as an inhibition in one case (intermediate, closer to bilingual mode) together with remaining priming effect or as an inactive state of the other (non-target) lexicon in another case (closer to monolingual mode). As we used inter-lingual homographs in our study, it would be interesting to investigate in a future study the effect of L2 interference during L1 processing, when there is absolutely no cue about L2 presence in the experiment to see whether it is possible to keep for example a highly proficient or even balanced bilingual in completely monolingual mode. Together with the monitoring of the subject’s language mode (one, although conscious, way of doing it can be explicit questioning about their perception of the experiment), it might become a new direction for investigating language interference in a completely monolingual context.

## Conclusion

Our both behavioral and electrophysiological results show that subject’s current language mode can influence the selectivity of his/her lexical processing. In the case of complete or partial non-selectivity, the mechanism of L2 interference when processing L1 is task-driven inhibition with some remaining priming effect from L2, reflected by partial decrease in N400 amplitude in response to the homograph-unrelated compared to control-unrelated group. In monolingual mode, when lexical processing is more selective than non-selective, the ‘non-active’ language is inactive rather than inhibited. This was reflected by the N400 amplitude in response to homograph-unrelated group similar to the one in response to the control-unrelated group, as well as by a small difference between the N400 potentials for the control-related and the homograph-related groups in response to a small difference in the word association strengths of the corresponding stimuli.

We showed that, when using sufficient distractors and appropriate conditioning, it is feasible to keep the subject closer to monolingual mode, even when inter-lingual homographs are present. Furthermore, we showed the importance to monitor the subject’s language mode during the experiment, e.g., via a post-hoc questioning of the subjects about their perception of experiment and whether they can assess the role of second language knowledge in the experiment. Given the importance of the issue, we advocate that bilingual lexical access models should take into account the subject’s current language mode, as it can support the interpretation of experimental results.

## Appendix

### Appendix A: Results with inclusion in analysis unfamiliar words and wrong behavioral responses

We independently analyzed the ERP data without rejecting trials with wrong responses and trials containing unfamiliar homographs. The results changed for the short experimental version only. The N400 amplitude in homograph unrelated group became even smaller and there was no significant difference between the N400 amplitudes of homograph unrelated and two related groups. Control unrelated group was the only stimulus group with significantly larger N400 amplitude compared to the other groups. The p values are as follows: 0.002 for homograph unrelated, and <0.0001 for control and homograph related groups.

Interestingly, such tendency was not observed for the long version, as the results remained the same. This can serve as another proof of our assumption that subjects of the long experimental version were closer to monolingual end of the language mode continuum, hence, they did not activate the English (L2) lexicon to the level that it could have a significant effect on the results, even when wrong answers were included in the analysis.

### Appendix B: Calculation of effect size

We used the following formula [49]:

ES= (μ1−μ2)stdpooled (B1)

where, *μ*1 is the averaged N400 amplitude in response to the stimulus group of interest,

*μ*2 is the averaged N400 amplitude in response to control unrelated stimulus group, and

*std*_pooled_ is the pooled standard deviation of both groups:

stdpooled= ((N1−1)std12+(N2−1)std22)(N1+N2−2) (B2)

with *N*_1_ the number of trials of group of interest,

*N*_2_ the number of trials of control unrelated group,

*std*_1_ the standard deviation of *μ*1

*std*_2_ the standard deviation of *μ*2

## Supporting Information

S1 Table Target stimulus list of the experiment. (DOCX)
