# Supplementary material for: ERP Response Unveils Effect of Second Language Manipulation on First Language Processing
Source: PLoS One. 2016 Nov 28;11(11):e0167194. doi: 10.1371/journal.pone.0167194 (PMC5125703; doi:10.1371/journal.pone.0167194)
Supplement: S1 Table — (DOCX) [file pone.0167194.s001.docx]

**S1 Table. Target stimulus list of the experiment**

| **Prime** | **target** | **Stimulus Group** |
| --- | --- | --- |
| star | zon | Homograph unrelated |
| stage | toestel | Homograph unrelated |
| brand | naam | Homograph unrelated |
| steel | ijzer | Homograph unrelated |
| sage | Verstandig | Homograph unrelated |
| stem | blad | Homograph unrelated |
| boot | Voet | Homograph unrelated |
| pit | Gat | Homograph unrelated |
| lap | Dans | Homograph unrelated |
| folder | bewaren | Homograph unrelated |
| angel | Vleugel | Homograph unrelated |
| boon | geschenk | Homograph unrelated |
| stout | gedrongen | Homograph unrelated |
| slot | machine | Homograph unrelated |
| record | speler | Homograph unrelated |
| beer | alcohol | Homograph unrelated |
| brief | Kort | Homograph unrelated |
| room | Bed | Homograph unrelated |
| roof | plafond | Homograph unrelated |
| list | Item | Homograph unrelated |
| worst | mooist | Homograph unrelated |
| pink | meisje | Homograph unrelated |
| pet | Poes | Homograph unrelated |
| ramp | rolstoel | Homograph unrelated |
| fee | Prijs | Homograph unrelated |
| spot | Hond | Homograph unrelated |
| kin | familie | Homograph unrelated |
| glad | gelukkig | Homograph unrelated |
| rug | tapijt | Homograph unrelated |
| arts | ambachten | Homograph unrelated |
| angel | Bij | Homograph Related |
| room | Melk | Homograph Related |
| spot | Licht | Homograph Related |
| star | Stijf | Homograph Related |
| worst | vlees | Homograph Related |
| pet | hoofd | Homograph Related |
| ramp | tsunami | Homograph Related |
| brand | Vuur | Homograph Related |
| lap | Stof | Homograph Related |
| stage | school | Homograph Related |
| sage | verhaal | Homograph Related |
| pink | vinger | Homograph Related |
| slot | sleutel | Homograph Related |
| rug | Pijn | Homograph Related |
| stem | geluid | Homograph Related |
| roof | diefstal | Homograph Related |
| stout | Kind | Homograph Related |
| list | Sluw | Homograph Related |
| arts | dokter | Homograph Related |
| record | sport | Homograph Related |
| folder | reclame | Homograph Related |
| kin | gezicht | Homograph Related |
| steel | borstel | Homograph Related |
| boon | groente | Homograph Related |
| fee | sprookje | Homograph Related |
| brief | schrijven | Homograph Related |
| glad | Ijs | Homograph Related |
| pit | appel | Homograph Related |
| boot | water | Homograph Related |
| beer | Bruin | Homograph Related |
| bloem | Roos | Control related |
| bakker | brood | Control related |
| regen | Nat | Control related |
| schoenen | veters | Control related |
| herfst | kleuren | Control related |
| dood | Kist | Control related |
| bellen | Deur | Control related |
| geloven | God | Control related |
| nacht | sterren | Control related |
| paal | Hout | Control related |
| Wieg | Baby | Control related |
| bril | lenzen | Control related |
| handdoek | afdrogen | Control related |
| schilderij | Verf | Control related |
| agressie | geweld | Control related |
| moeder | vader | Control related |
| maand | Jaar | Control related |
| lepel | Soep | Control related |
| stoel | zitten | Control related |
| voetbal | Bal | Control related |
| Oren | luisteren | Control related |
| sjaal | Koud | Control related |
| gooien | smijten | Control related |
| zak | winkel | Control related |
| plafond | Hoog | Control related |
| zwart | donker | Control related |
| pagina | bladzijde | Control related |
| Wolken | Lucht | Control related |
| trui | Wol | Control related |
| pijn | wonde | Control related |
| handdoek | Sap | Control unrelated |
| geloven | Vork | Control unrelated |
| dood | Varken | Control unrelated |
| pen | vliegtuig | Control unrelated |
| bril | soldaat | Control unrelated |
| herfst | Hond | Control unrelated |
| moeder | majoor | Control unrelated |
| Wolken | Wassen | Control unrelated |
| plafond | juweel | Control unrelated |
| bloem | Plaat | Control unrelated |
| Voetbal | schieten | Control unrelated |
| regen | Haar | Control unrelated |
| trui | troon | Control unrelated |
| gooien | zolder | Control unrelated |
| bellen | Schaal | Control unrelated |
| pijn | woning | Control unrelated |
| zak | zucht | Control unrelated |
| nacht | recht | Control unrelated |
| paal | Thee | Control unrelated |
| pagina | jongen | Control unrelated |
| oren | munt | Control unrelated |
| sjaal | Kers | Control unrelated |
| stoel | miljoen | Control unrelated |
| maand | Kust | Control unrelated |
| Schoenen | Vlinder | Control unrelated |
| agressie | Boel | Control unrelated |
| zwart | lippen | Control unrelated |
| wieg | kledij | Control unrelated |
| schilderij | Geld | Control unrelated |
| bakker | keuken | Control unrelated |
